# Prediction of miRNA-mRNA associations in Alzheimer’s disease mice using network topology

**DOI:** 10.1186/1471-2164-15-644

**Published:** 2014-08-03

**Authors:** Haneul Noh, Charny Park, Soojun Park, Young Seek Lee, Soo Young Cho, Hyemyung Seo

**Affiliations:** Department of Molecular & Life Sciences, Hanyang University, 1271 Sa-dong, Sangrok-gu, Ansan, Gyeonggi-do South Korea; Ewha Research Center for Systems Biology (ERCSB), Ewha Womans University, Seoul, 120-750 Korea; Bio-Medical IT Convergence Research Department, ETRI, 218 Gajeong-ro, Yusoeng-gu, Daejeon 305-700 Korea; MRC Harwell, Mammalian Genetics Unit, Harwell Science and Innovation Campus, Didcot, Oxfordshire OX11 0RD UK; Interdisciplinary Program for Bioinformatics, Program for Cancer Biology and BIO-MAX Institute, Seoul National University, Seoul, 151-742 Korea; Laboratory of Developmental Biology and Genomics, College of Veterinary Medicine, Research Institute for Veterinary Science BK21, Program for Veterinary Science, Seoul National University, Seoul, 151-742 Korea

**Keywords:** Alzheimer’s disease, microRNA, Transcriptome, Network module

## Abstract

**Background:**

Little is known about the relationship between miRNA and mRNA expression in Alzheimer’s disease (AD) at early- or late-symptomatic stages. Sequence-based target prediction algorithms and anti-correlation profiles have been applied to predict miRNA targets using omics data, but this approach often leads to false positive predictions. Here, we applied the joint profiling analysis of mRNA and miRNA expression levels to Tg6799 AD model mice at 4 and 8 months of age using a network topology-based method. We constructed gene regulatory networks and used the PageRank algorithm to predict significant interactions between miRNA and mRNA.

**Results:**

In total, 8 cluster modules were predicted by the transcriptome data for co-expression networks of AD pathology. In total, 54 miRNAs were identified as being differentially expressed in AD. Among these, 50 significant miRNA-mRNA interactions were predicted by integrating sequence target prediction, expression analysis, and the PageRank algorithm. We identified a set of miRNA-mRNA interactions that were changed in the hippocampus of Tg6799 AD model mice. We determined the expression levels of several candidate genes and miRNA. For functional validation in primary cultured neurons from Tg6799 mice (MT) and littermate (LM) controls, the overexpression of ARRDC3 enhanced PPP1R3C expression. ARRDC3 overexpression showed the tendency to decrease the expression of miR139-5p and miR3470a in both LM and MT primary cells. Pathological environment created by Aβ treatment increased the gene expression of PPP1R3C and Sfpq but did not significantly alter the expression of miR139-5p or miR3470a. Aβ treatment increased the promoter activity of ARRDC3 gene in LM primary cells but not in MT primary cells.

**Conclusions:**

Our results demonstrate AD-specific changes in the miRNA regulatory system as well as the relationship between the expression levels of miRNAs and their targets in the hippocampus of Tg6799 mice. These data help further our understanding of the function and mechanism of various miRNAs and their target genes in the molecular pathology of AD.

**Electronic supplementary material:**

The online version of this article (doi:10.1186/1471-2164-15-644) contains supplementary material, which is available to authorized users.

## Background

Alzheimer’s disease (AD) is the most common neurodegenerative disease worldwide and is characterized by cognitive dysfunction and neuronal degeneration [1]. Several pathological markers define AD, including altered amyloid beta (Aβ) production, Aβ aggregation, hyperphosphorylated tau, neurofibrillary tangles (NFTs) and senile plaques [2]. Microtubule-associated protein tau has been implicated in tangles and malfunction of cytoskeleton-related trafficking [3]. Aβ derived from amyloid precursor protein (APP) is involved in up-regulation of protein aggregates and related dysfunction in APP processing resulting from mutations in APP, presenillin1 (PSEN1) or presenillin2 (PSEN2) for early-onset AD and apolipoprotein E (APOE) for late-onset AD [4].

Omics approaches based on microarray and Next generation sequencing (NGS) technologies have been used to study the pathological mechanism of AD [5]. Genome-wide integrative approaches identify functional drivers of the disease and predict their specific regulatory functions. Using anti-correlation or target prediction programs, many studies have focused on the prediction of target gene or miRNA function; however, this approach has drawbacks because it may deliver false positive interactions and exhibit high noise ratios [6].

In this study, we hypothesized that simultaneous genome-wide analysis of mRNA and miRNA expression in an AD model at different disease stages will reveal differential gene expression patterns in AD, which may help to predict stage-dependent gene markers in AD pathology. To this end, we used Tg6799 transgenic mice, which contain mutant human APP (695) with Swedish (K670N, M671L), Florida (I716V), and London (V717I) Familial Alzheimer’s Disease (FAD) mutations, as well as human PS1 with M146L and L286V FAD mutations [7]. This AD transgenic model has been reported to show AD-like phenotypes such as rapid accumulation of Aβ, reduced levels of synaptic marker protein expression, increased p25 levels, neuronal loss, and memory impairment [7].

## Results and discussion

### Module prediction for AD

Using microarray data, we identified three modules that were closely related with AD. The function of each module was identified by the GO enrichment test of DAVID. The miRNA-mRNA interactions in AD were ranked by their topological importance through our co-expression network analysis and the ranking scores were applied to the PageRank algorithm.

**Figure 1:**
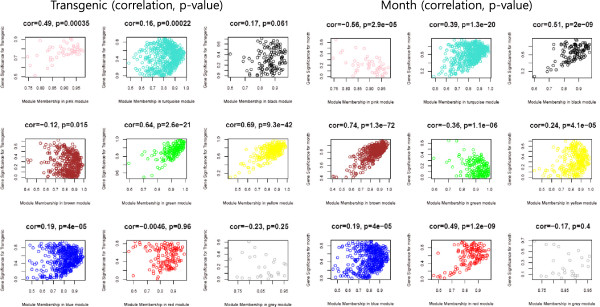
**Gene significance scatter plot for several gene modules.** Left panel represents gene scatter plot for the modules with genetic correlations and right panel represents the modules with age-related correlations. The statistically significant relationship between gene expression and genetic mutation or age was evaluated by Pearson Correlation.

In total, 2,197 differentially expressed genes (DEG) were identified and 8 modules were predicted from the expression microarray data. Modules and maturation factors were predicted using the Pearson Correlation method (Figure 1). The significance of each module was calculated using the WGCNA package based on gene significance (GS) and module membership (MM). Three modules showed positive correlations with AD (highlighted in green, pink, and yellow in Figure 1).

The green module showed positive correlation (cor: 0.64, p: 2.6e-21) with AD for sodium ion transport (Additional file 1: Table S1). AD has been reported to be associated with impaired glutamate clearance and depressed Na^+^/K^+^ ATPase levels in the brain [8]. Several solute carrier family members were found in the green module (i.e., SLC12a7, SLC12A2, SLC13A4, SLC22A5 and SLC4A5).

The pink module showed correlations with AD (cor: 0.49, p: 3.5e-4) and disease stage (cor: −0.56, p: 2.9e-05). Genes related to ion homeostasis and immune responses were identified in the pink module (Additional file 1: Table S1). Metal ion dyshomeostasis is a well-recognized cofactor in several neurodegenerative disorders [9, 10]. In AD, the process of Aβ misfolding and plaque aggregation is greatly influenced by alterations in the homeostasis of the aforementioned metal ions [11]. A strong genetic association between Aβ formation and AD has been reported in several studies [12] and we found that Aβ is highly expressed in the AD mouse model used in this study.

The yellow module was correlated with AD (cor: 0.69, p: 9.3e-42) and included genes related to cell death, apoptosis, and development (Additional file 1: Table S1). AD is correlated with region-specific neuronal cell loss [13, 14]. Notch 2, found in the yellow module, is known to regulate the Notch signaling pathway, which is involved in controlling the fate of neural and non-neural cells in development process and adults [15]. Several observations suggest that enhanced Notch signaling and expression could be instrumental in neurodegeneration in AD [16]. Huntingtin interacting protein 1 (HIP1), found in the yellow module, is related to cell death through caspase-3 activation in immortalized hippocampal neuroprogenitor cells [17]. Thus, the functions of the proteins in the green, pink, and yellow modules are directly associated with AD.

The black, brown and red modules showed relationships with disease stage and are involved in brain development including central nervous system development, neurogenesis, and forebrain development (Additional file 1: Table S1). The blue and turquoise modules showed significant relationships with AD and disease stage. The gene list for each of the 8 modules and the GSA results are presented in Additional file 1: Table S1 and Additional file 2: Table S2.

### miRNA-mRNA regulatory network in Tg6799 mice

We constructed a co-expression network from the functional gene module to study miRNA-mRNA interactions and the regulation of gene expression by miRNA. We integrated several approaches to predict miRNA targets and module regulators, which were predicted using 3-target site analysis. Although black, brown, and yellow modules did not show direct correlations with genetic AD (Figure 1), these 3 modules were considered as AD related miRNA modules because dysregulation of neurogenesis often cause neurodegenerative diseases. Using all these 6 modules (black, brown, green, pink, red, and yellow), we calculated all the pair expression correlations from target relations using sequence-based target prediction results and selected 254 miRNA-mRNA interactions by anti-correlation.

**Figure 2:**
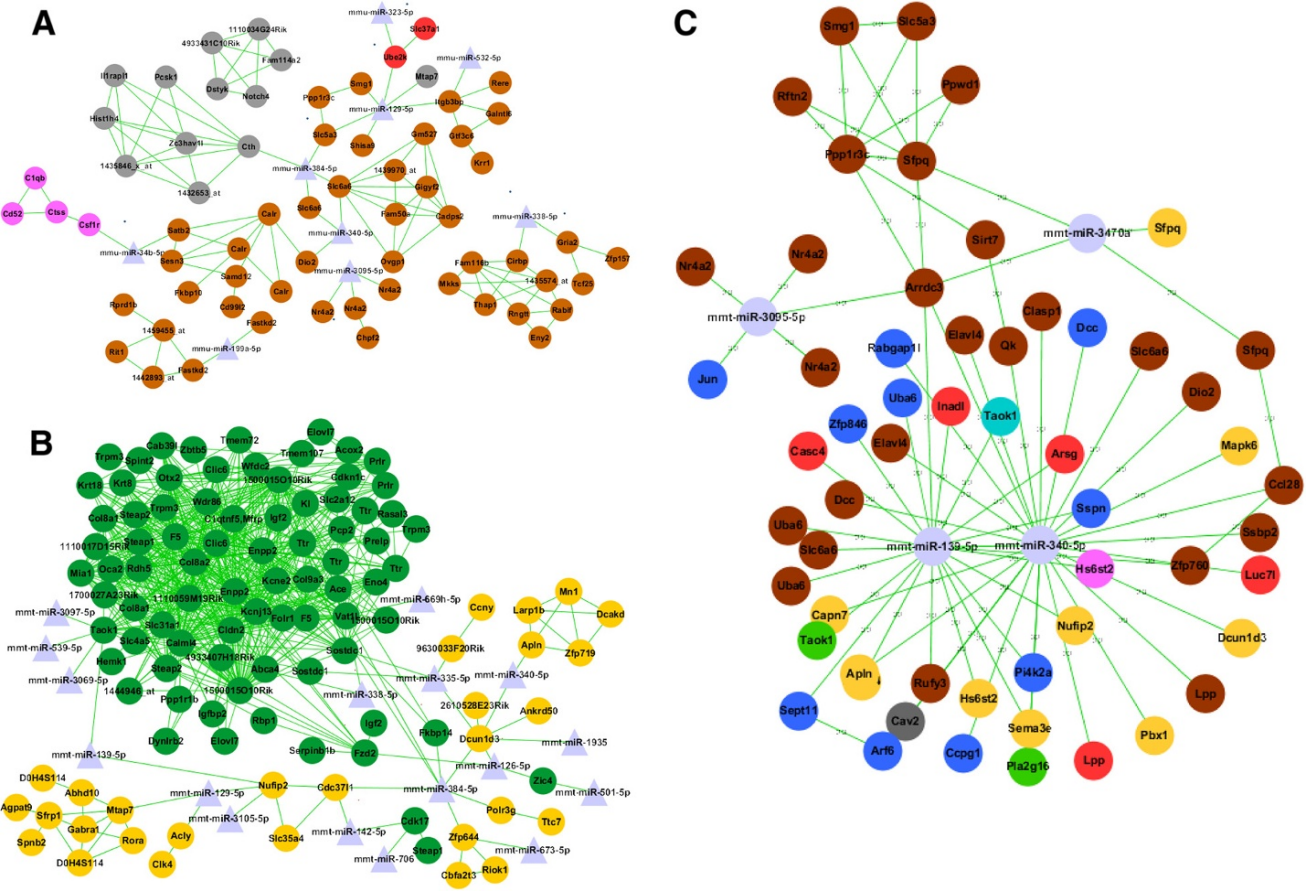
**The subnetwork of weighted coexpression networks of miRNA-mRNA in AD. (A)** The subnetwork of brown, grey, and pink module genes and related miRNAs. As presented in Figure 1 transgenic plots (left), these 3 modules show negative correlations. **(B)** The subnetwork of green (cor = 0.64) and yellow (cor = 0.69) module genes and related miRNAs. **(C)** The subnetwork of weighted coexpression networks. A compact network was constructed from arrestin domain containing 3 (ARRDC3) neighbors and related miRNAs to their target genes. The interaction between miRNA and their target genes were predicted by sequence analysis and expression anti-correlation.

The constructed co-expression weighted network (Figure 2) contains the black, brown, red, and pink modules. mRNAs in the red module correlate significantly with disease stage (correlation p-value ≤ 1.2e-09). miR-34b, which interacts with genes in the brown and pink modules, has been proposed as a key regulator to integrate age-associated diseases in the vertebrate brain [18–20]. It closely interacted with mRNAs in the brown and pink modules. Some genes in the disease stage-related modules have been reported to be associated with AD. For example, the members of the black and red modules are related to neurogenesis in normal developmental stages. The AD network was constructed using the yellow and green modules (Figure 2). In particular, miR-142 and miR-338 are known to be down-regulated in AD patients [21].

Significant interactions of miRNA-mRNA were predicted by the co-expression network. We assigned priority to miRNA-mRNA targets based on network topology. We assumed that highly ranked genes in miRNA-mRNA networks are more likely to play critical roles in the pathophysiology of AD. Using the PageRank algorithm, which is useful for describing the relative node importance in a network connection, we measured the PageRank score for miRNA-mRNA interactions and assigned the rank of interaction priority using only differentially expressed miRNAs (DEmiR) and DEG. We determined that 50 miRNA-mRNA interactions were significant targets and confirmed 19 miRNAs with a high PageRank score of <0.001 (Additional file 3: Table S3).

Microglia induction in AD can be explained by our miRNA-mRNA interaction prediction system. Our results predicted that miR-34b was involved in the regulation of colony stimulating factor 1 receptor (CSF1R), which has been reported to be critical for survival and proliferation of microglia [22]. Our microarray data showed that CSF1R was significantly down-regulated. Aging and neurodegeneration seem to be modulated by miR-34 expression through microglial activation [23, 24] and activation of microglial chemokine receptor 1 (CX3CR1) is known to lead to neuronal death in AD. Our current microarray data demonstrate that expression of Chemokine ligand 1 (CX3CL1) and ADAM metallopeptidase domain 10 (ADAM10) increased in Tg6799 mice at 8 months of age. ADAM10 is a predicted target of miR-129 and miR-448 and is involved in CX3CL1 cleavage. Cleavage of CX3CL1 by ADAM10 can lead to microglial activation of neuronal degeneration [25].

### Differential expression of miRNA in Tg6799 mice

In total, 54 differentially expressed (DE) miRNAs were identified in the early- and late-stage AD mouse model (cutoff FDR 0.1). We identified several miRNAs that correlated with AD (Additional file 4: Table S4). For example, miR-134, which is down-regulated in the brains of AD mice, is specifically expressed in the brain and has been demonstrated to negatively regulate dendritic spine formation *in vitro* [26]. miR-138 is enriched at synapses and modulate synaptic development and spine size through the control of acyl-protein thioesterase-1 (APT1) [27] and the depalmitoylation of G protein alpha 13 (GALPHA13), a down-stream target of APT1, which is an activator of Rho down-stream of G-protein coupled receptor (GPCR) [28]. miR-146b is down-regulated in AD mice and has been reported to be consistently altered in both hippocampus and medial frontal gyrus in AD mouse models [21]. miR-146b could be inferred as a negative regulator of innate immunity and its down-regulation in the AD brain provides support for an induction of Toll like receptor (TLR) signaling in AD [21]. miR-222 is highly expressed in the cortex during early fetal development and is related to the growth spurt in the cerebellum compared to the cortex [29]. miR-26a was observed to be down-regulated in Tg6799 mice at 4 months of age. miR-26a has been reported to be down-regulated in nasopharyngeal carcinomas and to suppress cell proliferation and colony formation by inducing G1-phase cell-cycle arrest [30]. miR-34b and miR-34c were up-regulated in our AD model mice and have been reported to be up-regulated in AD patients [18] (Additional file 4: Table S4).

**Figure 3:**
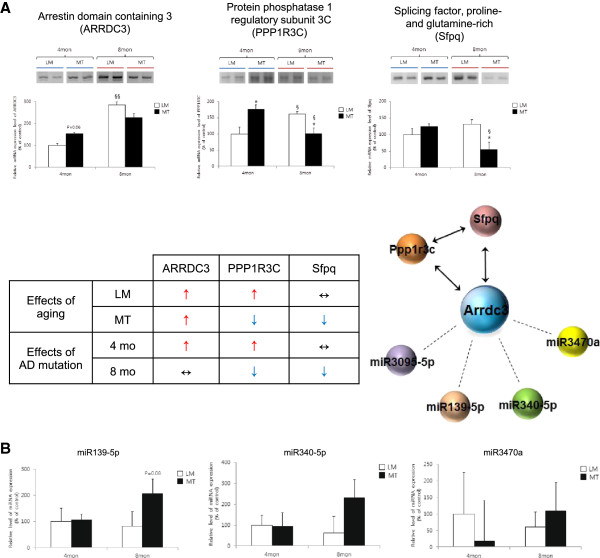
**Verification of the expression of mRNA and miRNA in the hippocampus of Tg6799 mice. (A)** The mRNA levels of ARRDC3, protein phosphatase 1 regulatory subunit 3C (PPP1R3C) and splicing factor, proline- and glutamine-rich (Sfpq) in the hippocampus of Tg6799 mice were verified using RT-PCR. (*: p < 0.05 within littermate (LM) or mutant Tg6799 (MT) mice; §: p < 0.05 between LM or MT mice; §§: p < 0.01 between LM or MT mice). **(B)** The expression levels of miR139-5p, miR340-5p, and miR3470a in the hippocampus of LM or MT mice were determined using real-time PCR.

We validated the expression levels of three miRNAs (miR139-5p, miR340-5p, and miR3470a) from the list of candidates. Real time-PCR results showed that at 8 months of age, miR139-5p expression showed tendency to increase (not significant, p = 0.08), in the hippocampi of Tg6799 mice compared to littermate controls (Figure 3). The expression of miR340-5p and miR3470a did not increase significantly in mutant Tg6799 mice at either 4 or 8 months of age.

### Differential expression of mRNA in Tg6799 mice: ARRDC3, PPP1R3C, Sfpq and metabolic dysfunction

Using integration analysis, we predicted functions of mRNA and miRNA associations in AD pathology. Focusing on that many neurodegenerative diseases are known to be caused by metabolic dysfunction, we decided to validate our prediction system through bio-energy metabolism related genes such as arrestin domain containing 3 (ARRDC3), a predicted target of miR-340, miR-139, miR-3095 and miR-3470a, in the brown module (Figures 3 and 2C). To validate the network topology analysis system, we selected significant interactions in non-DEmiR and determined the target gene expression. The mRNA level of ARRDC3 was increased in the hippocampus of Tg6799 (MT) mice at 4 months of age compared to their littermate (LM) controls. The mRNA level of ARRDC3 was significantly increased in an age-dependent manner in the hippocampus of LM controls (Figure 3). ARRDC3 mRNA also showed an age-dependent increase in MT mice. Interestingly, we determined that protein phosphatase 1 regulatory subunit 3C (PPP1R3C) exhibited direct relationships with age or AD mutation in consequence map data (Figure 3). The mRNA levels of PPP1R3C increased with age in LM controls and had a tendency to increase in MT mice at 4 months of age compared to LM controls (Figure 3; indicated as a red arrow). We determined that mRNA levels of PPP1R3C and splicing factor proline and glutamate-rich (Sfpq) decreased with age in MT mice and also decreased in MT mice at the late symptomatic stage compared to LM controls (Figure 3; indicated as a blue arrow).

**Figure 4:**
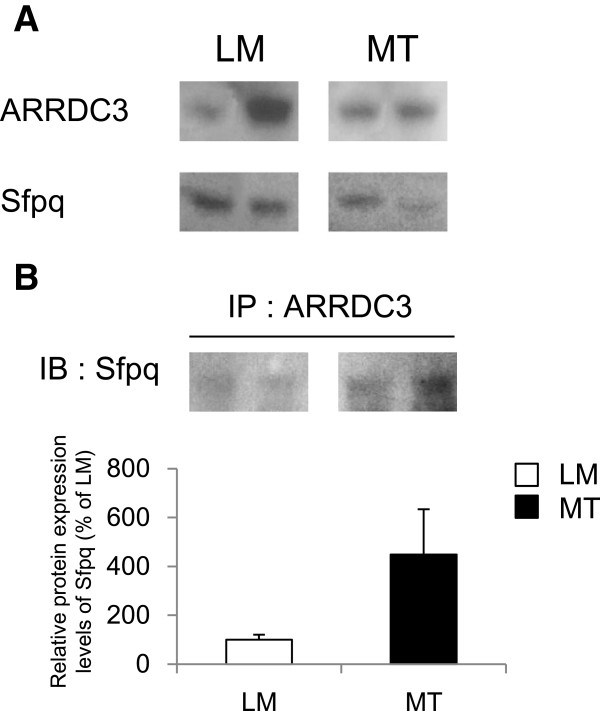
**Molecular interaction between ARRDC3 and Sfpq proteins. (A)** The protein levels of ARRDC3 and Sfpq in the hippocampus of Tg6799 mice were determined using Western blot. **(B)** The molecular interaction between ARRDC3 and Sfpq proteins was detected by co-immunoprecipitation experiment.

We next determined ARRDC3 and Sfpq protein expression levels by performing semi-quantitative Western blot analysis using the hippocampus samples from MT mice and LM controls at 10 months of age. The protein expression levels of ARRDC3 and Sfpq decreased in MT mice compared to LM controls (Figure 4). Co-immunoprecipitation data showed direct molecular interaction between ARRDC3 and Sfpq proteins and this interaction was increased in MT mice compared to LM controls (Figure 4).

ARRDC3 has been implicated in the regulation of body mass and energy metabolism and plays a role as a tumor suppressor [31–33]. As a down-stream target of ARRDC3, Sfpq has been implicated in cellular processes such as transcriptional regulation and RNA splicing, both of which are related to circadian rhythms. Sfpq is expressed in neurons and glia and is confined to the nuclei in both cell types [34]. PPP1R3C could be induced by hypoxia as a hypoxia induced factor (HIF)-targeted gene. Protein phosphatase 1 (PP1) is known to target glycogen (PTG) and to regulate glycogen metabolism [35]. ARRDC3, PPP1R3C, and Sfpq belong to a metabolism-related category of genes, as we demonstrated in the brown module. Previously, Poll et al. demonstrated that arrestin-dependent GPCR signaling is controlled by rapid dephosphorylation of PP1 [36].

**Figure 5:**
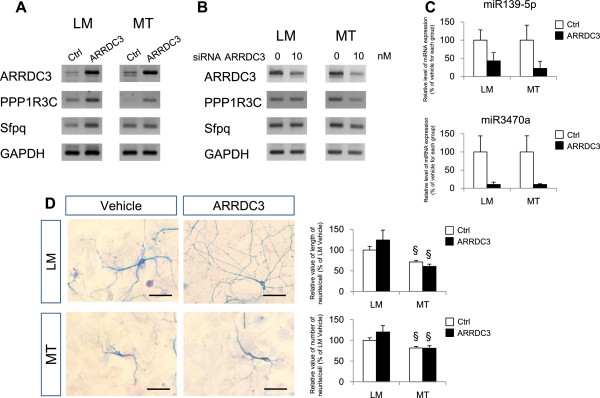
**Validation of ARRDC3 function in primary cultured cortical neurons from Tg6799 transgenic mice. (A)** Overexpression of ARRDC3 gene in primary cultured cortical neurons from Tg6799 mice. RT-PCR results show that the mRNA levels of PPP1R3C and Sfpq were increased by ARRDC3 overexpression. **(B)** Knock-down of ARRDC3 gene in primary cultured cortical neurons from Tg6799 mice. The mRNA levels of PPP1R3C and Sfpq were decreased by the treatment of ARRDC3 siRNA. **(C)** Real-time PCR data show that the expression levels of miR139-5p and miR3470a had decreasing tendency in the primary cortical neurons of Tg6799 mice. **(D)** Immunocytochemical staining show no significant enhancement in the number and length of neurites in ARRDC3 overexpressed primary cortical neurons from Tg6799 mice (§: p < 0.05 between LM and MT mice).

For functional validation of ARRDC3, we overexpressed ARRDC3 by transfection or silenced ARRDC3 expression with siRNA in primary cultured cortical neurons from Tg6799 (MT) mice and littermate (LM) controls. ARRDC3 overexpression increased the mRNA levels of ARRDC3, PPP1R3C, and Sfpq in the cortical neurons of both LM and MT mice (Figure 5A). After silencing with ARRDC3 siRNA, the mRNA levels of PPP1R3C and Sfpq decreased in MT cortical neurons (Figure 5B). Real time-PCR results showed that miR139-5p and miR3470a expressions have tendency to decrease after ARRDC3 overexpression in both LM and MT primary cortical neurons (Figure 5C). We also determined the length and the number of neurites in primary cultured neurons from Tg6799 mice using anti-tau antibody. The number of tau-positive neurons from MT mice significantly decreased and the length of neurites decreased as well compared to LM primary neurons. However, ARRDC3 overexpression could not alter the length or the number of neurites compared to vehicle treated MT neurons (Figure 5D).

**Figure 6:**
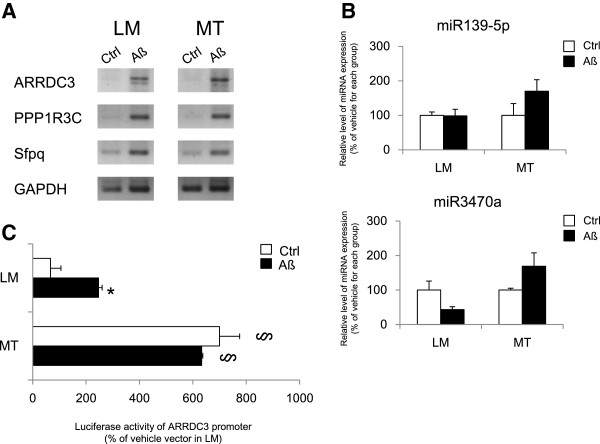
**Determination of the expression of ARRDC3 related mRNA/miRNA under A administration. (A)** RT-PCR show that the mRNA levels of ARRDC3, PPP1R3C and Sfpq were increased by Aβ administration in primary cultured cortical neurons of Tg6799 mice. **(B)** Real time PCR show that the expression levels of miR139-5p and miR3470a were not significantly altered by Aβ administration in primary cortical neurons from Tg6799 mice. **(C)** Luciferase activity of ARRDC3 promoter increased after Aβ treatment in LM cortical neurons but not in MT neurons.

To determine ARRDC3, Sfpq, and PPP1R3C mRNA levels and miRNA interaction in Aβ toxic condition, we administered 2 μM Aβ into the primary cultured cortical neurons from Tg6799 mice. After Aβ treatment, the mRNA levels of ARRDC3, PPP1R3C, and Sfpq increased in the cortical neurons of both LM and MT mice (Figure 6A). Real time-PCR results showed that after Aβ treatment, miR139-5p and miR3470a expressions have tendency to increase in cortical neurons of Tg6799 mice compared to vehicle treated MT neurons (Figure 6B). To detect luciferase activity of ARRDC3 promoter in toxic condition, we transfected ARRDC3 promoter into primary cultured cortical neurons of Tg6799 mice then administered Aβ. Luciferase activity for ARRDC3 promoter significantly increased in MT cortical neurons compared to LM control (Figure 6C). Also after Aβ treatment, luciferase activity for ARRDC3 promoter significantly increased in LM cortical neurons compared to vehicle treatment (Figure 6C).

Taken together, these data suggest that ARRDC3, PPP1R3C, and Sfpq interact with each other in regulating the age- and disease-specific gene expression of similar biological pathways such as bioenergetics and metabolism, which are very strong candidate categories implicated in the pathophysiological progressive changes of neurodegeneration [37–40].

## Conclusions

Our results demonstrate AD-specific changes in the miRNA regulatory system and a relationship between the levels of miRNAs and their targets in the AD hippocampus. We identified AD-related modules and provided a specific network that integrates differential miRNA and mRNA expression profiles. We proposed relevant miRNA-mRNA target relationships in an AD based on network topological priorities and miRNA mediated mechanism in AD pathology. Our data highlight the functions and mechanisms of various miRNAs and their target genes in the molecular pathology of AD including the genes which are related in ion transport, ion homeostasis, immune responses, cell death, apoptosis, development, and bio-energy metabolism. Knowledge of candidate genes identified through the simultaneous genome-wide profiling of miRNA and mRNA may be useful for the development of future therapeutics in AD.

## Methods

### Animal Husbandry

Tg6799 transgenic mice were purchased from the Jackson Laboratory (Bar Harbor, ME) and were housed under a 12-h light–dark cycle with free access to food and water. Female Tg6799 mice (n = 8 in each group) were maintained until used for the experiment (4 months for early symptomatic stage and 8 months of age for late symptomatic stage). After breeding in an expansion colony, male Tg6799 transgenic mice were mated with female littermate mice. The genotype of each mouse was confirmed by PCR using the DNA from mouse tail tissue. The mice had free access to food and water. All animal studies were conducted in accordance with IACUC guidelines and were approved by the IACUC committee at Hanyang University (HY-IACUC-09-017).

### RNA Sample preparation

RNA samples were isolated from the hippocampus (HC) using TRI-Reagent (Sigma-Aldrich, St. Louis, MO) according to the manufacturer’s instructions. The total RNA pellet was dissolved in nuclease-free water and RNA quality and quantity were assessed by Agilent Bioanalyzer 2100 analysis (Agilent, Santa Clara, CA). RNA with RNA Integrity Number (RIN) above 8 was used for microarray and sequencing. Gene expression was analyzed with GeneChip® Mouse Genome 430 2.0 Arrays (Affymetrix, Santa Clara, CA), which comprises over 45,000 probe sets representing approximately 28,700 well-characterized mouse genes. For each gene, eleven pairs of oligonucleotide probes were synthesized in situ on the arrays.

### mRNA microarray hybridization and analysis

Biotinylated cRNA were prepared from 250 ng total RNA according to the standard Affymetrix protocol (Expression Analysis Technical Manual, 2001, Affymetrix). Following fragmentation, 15 μg of RNA were hybridized for 16 hr at 45°C on a GeneChip Mouse Genome Array. GeneChips were washed and stained in the Affymetrix Fluidics Station 450. GeneChips were scanned using the Affymetrix Gene Chip Scanner 3000 7G. Eight microarray samples were normalized by the RMA method of R affy package [41]. DEG testing was accomplished using the 4 class limma test of 2 mutants and 2 aging factors. Finally, 2,197 probes were considered to have significantly differential expression by a cut-off p-value < 0.05.

### miRNA sequencing and analysis

RNA quality was assessed by the absence of smear of 18S and 28S bands as analyzed by the Bio analyzer 2100 (Agilent Technologies, Santa Clara, CA). For the construction of micro RNA libraries, IonTotal RNA-Seq Kit v2 and Ion Xpress™ RNA-Seq Barcode kit (Life Technologies, Foster City, CA) were used. The Ion Total RNA-Seq Kit v2 (Life Technologies) was used for the preparation of micro RNA libraries according to the manufacturer’s instructions starting with 100 ng total RNA. The template was prepared from the libraries using the Ion OneTouch™ System (Life Technologies) and sequenced on an Ion PGM™ Sequencer (Life Technologies). Total read counts are shown in Additional file 5: Table S5. As a preprocessing step, the sequencing adapter was trimmed and read to be over the range of miRNA length (18–30 nucleotides) were excluded. We allowed bowtie2 with options of one mismatch and used mouse miRbase ver. 19 as a reference. [42, 43]. After alignment, a total of 527 miRNAs were identified. Raw level data from the miRNA read count were normalized using the quintile method and considered as miRNA expression. Differentially expressed miRNA (DEmiR) was also tested using limma-like microarray conditions. Finally 54 DEmiRs were detected within a cutoff p-value ≤ 0.1.

### Gene expression network for miRNA target integration

For the genome wide study, we detected gene set modules from DEG and built a weight co-expression network. The network was constructed using WGCNA R packages with 2,197 DEGs from the limma test [44]. We detected 9 modules with block-wise module function. To evaluate significant modules related to the sample traits of AD mutation and aging, we performed scatter plots and correlations of gene significance and MM were measured. MM is a quantitative measure for the correlation of the module eigengene with the gene expression profile. MM and correlation allow quantification of gene member similarity for each module. We selected 8 significant modules using these correlations. The grey module was removed because of the small gene set count (27 probes, 23 identified genes). Finally for constructing a weighted network, a topological overlap measure (TOM), as a measure of the robustness of the network interconnectedness, was calculated based on the adjacency matrix from the 8 module gene set. From module TOMs with a weighted network threshold 0.25, a network dataset was exported to Cytoscape. Functional enrichment of each module was verified using the DAVID database (p-value < 0.1) [45].

An integrative network of miRNAs and genes was constructed after miRNA-target gene prediction. miRNA and target relationships were predicted by both expressional and sequential analysis. All miRNAs were considered for miRNA-target anti-correlated expression. However, we considered and calculated correlations in view of sample subsets in AD factors or aging factors. Additionally, the sequence-based target relation was predicted using both TargetScan and miRAN.org [46, 47]. miRNA priority was calculated using the PageRank algorithm to consider topological importance [48]. From the sequence and expression-based approaches, we removed insignificant miRNA and target gene relationships and ranked miRNA importance by PageRank score. The raw data were uploaded into the gene expression omnibus (GEO) database with an accession number of GSE52023.

### RT-PCR

The SuperScript III First-Strand Synthesis System (Invitrogen, Carlsbad, CA) was used to synthesize first-strand cDNA from an equal amount (500 ng) of RNA. The following primer sequences were used for RT-PCR in this study: arrestin domain containing 3, 5′ -TTCTCAGTTTGCCCCTCGTC- 3′ (forward) and 5′ -TCCTCTGCAAACGTGTCTCC- 3′ (reverse); protein phosphatase 1, regulatory subunit 3, 5' -ACGATGGAAGTCCTTGGATG- 3' (forward) and 5' -TCCATGCGCCTTAATTCTTC- 3' (reverse); splicing factor proline/glutamine rich, 5' -ACGATGGAAGTVCTTGGATG- 3' (forward) and 5' - TCCATGCGCCCTTAATTCTTC- 3' (reverse); glyceraldehyde 3-phosphate dehydrogenase, 5′-AGAACATCATCCCTGCATCC- 3′ (forward) and 5′ -TCCACCACCCTGTTGCCTGTA- 3′ (reverse).

### Protein sample preparation

Protein samples were prepared from the hippocampus in a homogenization buffer (50 mM Tris-Cl (pH8.0), 150 mM NaCl, 5 mM EDTA, 1% Triton X-100) containing protease and phosphatase inhibitors: 10 μg/ml aprotinin, 25 μg/ml leupeptin, 10 μg/ml pepstatin, 10 μg/ml PMSF. Homogenates were centrifuged at 14,000 g for 30 min at 4°C and the supernatants were collected and stored at 80°C before use.

### Western blot

Equal amounts (10 μg) of protein sample were used for western blot analysis [49]. Protein samples were loaded in 10% sodium dodecyl surfate (SDS)–polyacrylamide gels and transferred to polyvinylidene difluoride (PVDF) membrane (BIO-RAD, Hercules, CA). The following antibodies were used for primary antibody reaction: polyclonal anti-ARRDC3 (1:2,000; Abcam, Cambridge, MA) and polyclonal anti-Sfpq (1:2,000; Abcam, Cambridge, MA) and for secondary antibody reaction: horseradish peroxidase (HRP)-linked anti-rabbit (1:2500; Vector, Burlingame, CA).

### Co-immunoprecipitation (Co-IP)

Equal amounts (10 μg) of protein samples were immunoprecipitated using polyclonal anti-ARRDC3 (1:2,000; Abcam, Cambridge, MA) at 4°C for overnight, followed by binding anti-ARRDC3 to protein A agarose (Sigma, St. Louis, MO). Immunoprecipitated protein expression was confirmed by immunoblotting with used polyclonal anti-Sfpq (1:2,000; Abcam, Cambridge, MA).

### Primary cell culture

Cortex and hippocampus were dissected from the brains of Tg6799 transgenic mice and their littermate controls at postnatal day 1 (p1). Neurons were cultured on glass cover slips or cell culture multi-well plates, both coated with poly-L-ornithine (Sigma, St. Louis, MO), fibronectin (Sigma, St. Louis, MO), and laminin (Sigma, St. Louis, MO). Culture media used was DMEM/F-12 with 10% fetal bovine serum (FBS, GenDEPOT, San Diego, CA), 100 U/ml penicillin and 100 μg/ml streptomycin (Sigma, St. Louis, MO), and 2 mM L-glutamin (Gibco, Carlsbad, CA) supplemented with 5% B-27 supplement (Gibco, Carlsbad, CA) and 10 ng/ml bFGF (Invitrogen, Carlsbad, CA) at densities of 3.63 × 10^4^ cells/cm^2^.

### Knock down or overexpression of ARRDC3

To analyze the role of ARRDC3, we transfected the primary cells with ARRDC3 cDNA on the 7^th^ day of primary cell culture. The mammalian expression vector pCMV6-XL4 was used for the overexpression of full-length human ARRDC3 cDNA (NM 020801.1) (Origene Technologies, Inc., Rockville, MD). The plasmid was transiently transfected with standard calcium-phosphate preparation. After 24 hr, transfected cells were administered with 2 μM Aβ for 24 hr. Next, we transfected the primary cells on the 7^th^ day of cell culture with ARRDC3 siRNA or scrambled ARRDC3 siRNA as negative control (data not shown). The sequence for ARRDC3 siRNA is 5′ -GACACCACUUGCUACCUCA- 3′ and the sequence for scrambled ARRDC3 siRNA is 5′ -ACACUCUAACCGGCUUACC- 3′. The siRNAs were transfected into the primary cells by Gene-mute siRNA Transfection Reagent (SignaGene laboratories, Gaithersburg, MD) for 48 hr, as described by the manufacturer.

### ARRDC3 promoter-reporter construction

DNA fragments (−280 bp) upstream of the ARRDC3 gene were PCR-amplified using human SHSY-5Y cell gDNA as template with following primers [50]: (−280 bp) 5' -GGGGTACCGGGGTTGTTTTTCACAAAGCTG- 3' (forward) and 5' -ATCTCGGTCTTCACCTTTCCCAGCACC- 3' (reverse). The PCR fragments were purified, digested, and cloned into the luciferase vector pGL3-Basic (Promega, Medison, WI) with KpnI and XhoI restriction sites.

### Transient transfection and luciferase assay

Transfection of ARRDC3 promoter was conducted into primary cells on the 7^th^ day of culture using the standard calcium-phosphate preparation. After 24 hr, transfected cells were administered with 2 μM Aβ for 24 hr, lysed, then measured for luciferase activity using TD-20/20 luminometer (Turner Designs, Sunnyvale, CA) by injecting 40 μl per tube of a buffer containing 100 mM Tris-Cl (pH 7.8), 15 mM MgSO_4_, 10 mM ATP, and 65 μM of luciferin. All transfections were carried out in triplicate.

### Immunocytochemistry

After treatment, the primary cells on coated cover slips were post-fixed in 4% paraformaldehyde for 10 min. The cells were blocked for 30 min then incubated with polyclonal tau (Tau46) antibody (1:500; Cell Signaling, Danvers, MA) for 3 hr. After secondary antibody incubation, the cells were stained with blue alkaline phosphate substrate solution (Vector Laboratories Inc., Burilngame, CA) then mounted on gelatin-coated slides for microscopic analysis. Images were obtained using a Carl Zeiss microscope (Axio observer, Oberkochem, Germany). The number of tau-positive cells was counted from microscopic images and analysis was performed blindly by two different assessors.

### Detection of miRNA expression

RNA samples were extracted from mouse hippocampal tissue using the miRCURY RNA Isolation Kit (Exiqon, Woburn, MA) according to the manufacturer’s protocol. cDNA was synthesized using the Universal cDNA synthesis kit II (Exiqon, Woburn, MA) and was used as a template for microRNA real-time PCR using ExiLENT SYBR Green master mix (Exiqon). The following primers were used for real-time PCR in this study: mmu-mir-139-5p, 5' -UCUACAGUGCACGUGUCUCCAG- 3'; mmu-mir-340-5p, 5' -UUAUAAAGCAAUGAGACUGAUU- 3'; mmu0mir-3470a, 5' -UCACUUUGUAGACCAGGCUGG- 3' and RNU5G control primer (Exiqon, Woburn, MA). Quantitative real-time PCR was performed for 10 min at 95°C followed by 10 sec at 95°C and 1 min at 60°C for 45 cycles. At the end of PCR cycles, melting curve analyses were performed for each PCR product.

### Statistical analysis

All statistical analyses were accomplished using SPSS (version 17; SPSS Inc., IL). Data were objectively compared using independent-sample T-tests. Differences between groups were considered statistically significant when the *p* value was less than 0.05.

### Availability of supporting data

The data sets supporting the results of this article are included within the article and its additional files. miRNA and mRNA microarray data have been deposited in GEO public repository with ID GSE52022 (http://www.ncbi.nlm.nih.gov/geo/query/acc.cgi?acc=GSE52022), ID GSE52023 (http://www.ncbi.nlm.nih.gov/geo/query/acc.cgi?acc=GSE52023), and ID GSE52024 (http://www.ncbi.nlm.nih.gov/geo/query/acc.cgi?acc=GSE52024).

## Electronic supplementary material

Additional file 1: Table S1: Gene Set Analysis results for modules. (XLS 476 KB)

Additional file 2: Table S2: Gene list for each module. (XLSX 162 KB)

Additional file 3: Table S3: List for PageRank scores. (XLSX 94 KB)

Additional file 4: Table S4: List of differentially expressed genes (DEG) and differentially expressed miRNAs (DEmiR). (XLSX 6 MB)

Additional file 5: Table S5: Statistics for small RNA-seq. (XLS 24 KB)
